# Five years of local control of subscapularis aggressive fibromatosis managed by surgery and imatinib: a case report

**DOI:** 10.1186/1752-1947-8-416

**Published:** 2014-12-09

**Authors:** Abou Dao, Nadia Benchakroun, Hamza Jabir, Amina Taleb, Zineb Bouchbika, Nezha Tawfiq, Hassan Jouhadi, Souha Sahraoui, Abdellatif Benider

**Affiliations:** Centre Mohammed VI pour le Traitement des Cancers, CHU Ibn Rochd, Casablanca, Morocco

**Keywords:** Aggressive fibromatosis, Imatinib, Local control, Recurrence, Subscapularis, Surgery

## Abstract

**Introduction:**

Imatinib, a tyrosine kinase inhibitor, is a major therapeutic option for the management of unresectable aggressive fibromatosis. Unfortunately, for most patients of low or very low average income countries, surgery often is the first treatment option. This is related to unavailability of chemotherapy or targeted therapy, and to a lack of financial resources or surgeons’ lack of knowledge of other therapeutic options.

**Case presentation:**

In 2010, a 26-year-old Moroccan man was referred to our oncology and radiotherapy center by his surgeon for the management of a recurrent tumor of his right subscapularis muscle. Before his assessment in our center, two resections were performed by his surgeon after performing an incision biopsy and magnetic resonance imaging. Postoperative magnetic resonance imaging was performed and showed a right axillary nodule size 2.1cm regarding a collection with a residual tumor. We decided to administer imatinib 400mg daily by mouth. Clinical and magnetic resonance imaging evaluation were performed regularly and reported a stable tumor. We reported no adverse side effects to imatinib regarding Common Terminology Criteria for Adverse Events grading.

**Conclusions:**

Recurrences are high during aggressive fibromatosis management. Systemic treatment with imatinib for unresectable or recurrent tumors with positive c-KIT could be the best therapeutic option. In our case report, the patient was stabilized with imatinib for 30 months and he had a very good quality of life.

## Introduction

Imatinib, a tyrosine kinase inhibitor, is a major therapeutic option for the management of unresectable aggressive fibromatosis (AF) or desmoid tumors [1]. These tumors are non-malignant and aggressive; they can occur anywhere in the body. Extra-abdominal forms are usually confined to the musculature and the overlying aponeurosis or fascia but the neoplasm may infiltrate the surrounding tissue up to 2 to 3cm outside the palpable tumor [2]. Management of these tumors is not standardized but relies on the combination of surgery, radiotherapy and/or systemic therapy. Local control is the main goal of treatment and there has been a change in the management of these tumors from aggressive surgical resection to function preservation [3]. The surgical resection rate regarding primary treatment modality for desmoid tumors when functionally and cosmetically acceptable with reported local control is 75 to 80% [3]. For surgery alone, local recurrence rates varied from 24 to 77% which justified the use of other therapeutic options. Systemic therapy has been reported regarding cytotoxic agents [4] but with documented cardiotoxicity and myelosuppression. Regarding the relative toxicities of cytotoxic agents, hormonal therapy and tyrosine kinase inhibitors are increasingly reported as therapeutic options [5]. Imatinib mesylate (Gleevec^®^) is a specific tyrosine kinase inhibitor highly used for targeting c-KIT, breakpoint cluster region-abelson gene (*BCR-ABL*), platelet-derived growth factor receptors (PDGFRs) and macrophage colony-stimulating factor receptor. Longtime disease stabilization with imatinib mesylate has been reported in different series of patients with relapsing desmoid tumors, with 1-year progression-free survival rates close to 60 to 70% [6–8]. Unfortunately, for most patients of low or very low average income countries, surgery often is the first treatment option. This is related to the unavailability of chemotherapy or targeted therapy, and a lack of financial resources or lack of surgeons’ knowledge of other therapeutic options. Although AF tumors are benign, the best management of AF is a multidisciplinary approach to plan local control with acceptable morbidity.

## Case presentation

**Figure 1:**
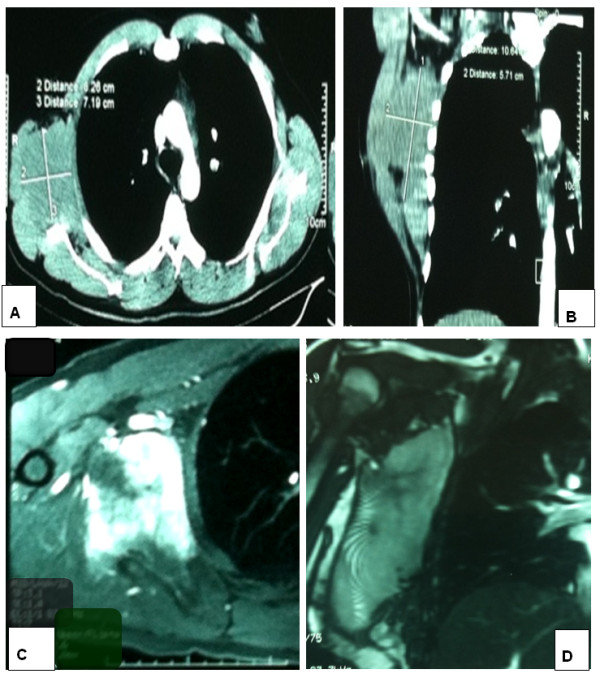
**Images of the first recurrence.** Thoracic computed tomography axial cut **(A)** and sagittal cut **(B)**. Auxiliary magnetic resonance imaging with axial **(C)** and sagittal **(D)** cut showing mass measuring 130mm. This mass was localized on the right chest wall, between scapula and chest wall, hypointensity T1, hypersignal T2; this mass displaces the muscle structures; auxiliary vessels are permeable.

**Figure 2:**
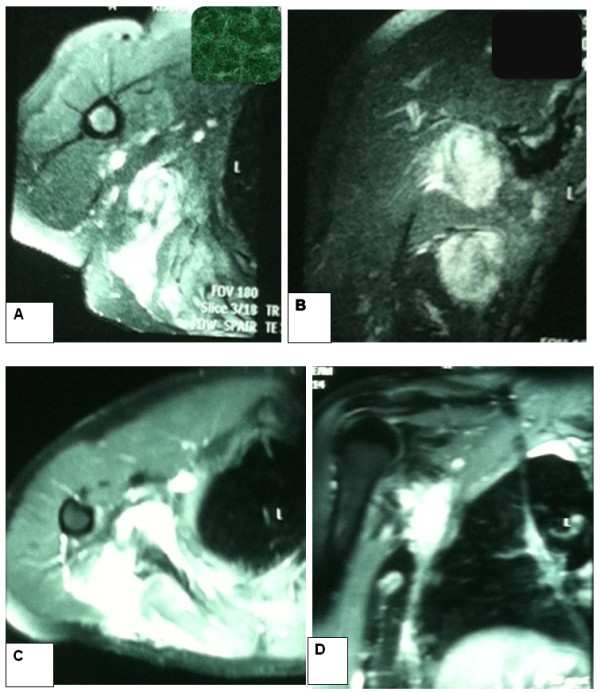
**Pre and post second surgery magnetic resonance imaging.** Axillary magnetic resonance imaging performed before second surgery **(A-B)** and after surgery **(C-D)**. Magnetic resonance imaging axial and sagittal cut **(A-B)** showing soft-tissue mass recurrences measuring 126mm on high axis in right chest wall, hypointensity T1 and hyperintensity T2, contact with axillary pedicle and brachial vessels homolateral. These vessels remains permeable and are not involved; a second similar soft tissue mass of right chest wall in posterior areas measuring 61mm in diameter was noted. After surgery, right auxiliary magnetic resonance imaging **(C-D)** was performed and showed a residual tumor measuring 21mm.

**Figure 3:**
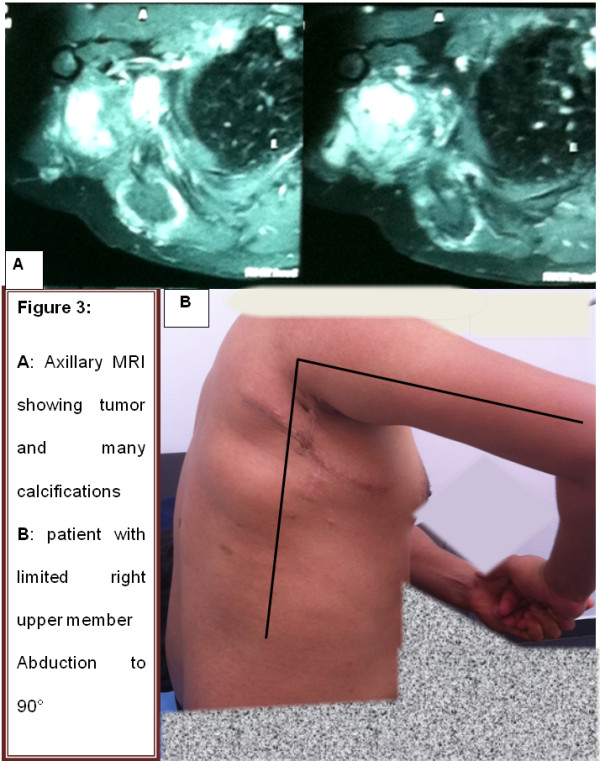
**Last magnetic resonance imaging and patient status.** Axillary magnetic resonance imaging **(A)** showing tumor and many intratumoral calcifications. **(B)** Patient with limited right upper member abduction to 90°.

In 2010, a 26-year-old Moroccan man was referred to our oncology and radiotherapy center for management of recurrent tumor of his right subscapularis. No pathological medical or surgical history was reported and no alcohol or tobacco habits. One year before, he had presented to his surgeon with a subscapularis tumor which had appeared gradually and increased in size during 6 months without associated pain or other symptoms. A physical examination reported a mass size 10cm, palpated in his right subscapularis region. A first resection was realized. Histopathological analysis demonstrated spindle-shaped cells with no identifiable nuclear pleomorphism or mitotic activity. There was no necrosis. A benign tumor with spindle-shaped cells is suspected. On immunohistochemical analysis, the cells stained positive for anti-smooth muscle actin, favoring a smooth muscle origin. The diagnostic of AF was retained. Unfortunately, the mass recurred within a period of 10 months and continued to increase in size reaching 13cm on a computed tomography scan and magnetic resonance imaging (MRI; Figure 1) without regional structures involved (bone, muscle or vascular). A second tumor and lymph nodes resection were performed by another surgeon who reported “a very hard resection without cleavage plane”. Histological analysis regarding two fragments size 4×3cm and 14×10×8cm led to a conclusion of AF tumor; hormonal receptor was not found. The resections of margins were narrowed and lymph node resections regarding four nodes were not involved. At assessment in our oncology and radiotherapy center after this second resection, his World Health Organization performance status was zero, weight 84kg, height 177cm. There was no induration or palpable mass. A MRI performed after the repeat surgery was normal. During follow up, a recurrence was suspected 1 year after the second resection. An axillary MRI (Figure 2A and 2B) was performed and a large mass was found in the last tumor site, measuring 12.6cm involving deltoid muscles and extending to axillary area. A third resection was realized and a histopathological examination showed the same AF tumor with a low positivity of c-KIT. Margins were narrowed. Post-surgery MRI (Figure 2C and 2D) was performed and showed a right axillary residual tumor measuring 2.1cm. Diagnosis of recurrent AF tumor with positive c-KIT and without hormonal receptor was retained. The decision to administrate imatinib 400mg daily by mouth was taken. His follow up was performed by clinical examination and was normal during 6 months. When we stopped imatinib administration during 1 month, the tumor grew to a size of 4cm. Retreatment with imatinib was decided. Clinical and MRI evaluation were performed regularly and they reported a stable tumor. The last MRI (Figure 3A) was performed in January 2014; it showed a stabilized tumor and many intratumoral calcifications. We reported no side effects regarding Common Terminology Criteria for Adverse Events. Currently he is feeling well but he continues to have ankylosis (90°) in his right upper limb due to the particular localization of AF and surgery (Figure 3B).

## Discussion

In low average income countries like Morocco, according to gross national income of World Bank Atlas 2008, targeted therapies are not always accessible to all patients. For this case report we presented to our patient the advantages and disadvantages of imatinib versus iterated surgeries. During management of our case report, despite major surgery, two recurrences were reported within 12 months which justified the introduction of imatinib. In the subscapularis region, which is an unusual localization of desmoid tumor, surgery was very hard and margins were almost involved or narrow despite the surgeon’s experience. Thus, the patient took imatinib during 2.5 years and he was stabilized with a good quality of life. Our patient received 400mg orally daily. The daily dose of imatinib was controversial in the literature. Some authors started with a standard dose [9], other authors started with a low dose and raised it after 2 weeks [10], and another author started with a high dose and decreased the dose when side effects occurred [7]. We reported no side effects within this case report. Imatinib is well tolerated in published series and only side effects of grade I/II tiredness and edema were reported without any major effects (grade III/IV) [7].

Mace *et al.* resumed the pharmaceuticals action of imatinib mesylate (Gleevec™; Novartis Pharmaceuticals, Hanover, NJ, USA) as a selective tyrosine kinase inhibitor targeting Bcr-Abl fusion protein in chronic myelogenous leukemia, multiple class 3 receptor tyrosine kinases including PDGFR-α and PDGFR-β, as well as the c-KIT subtype [7].

This agent blocks ligand-activated receptor phosphorylation and mitogen-activated kinase activation and proliferation, resulting in the inhibition of cellular growth and proliferation. Complete or even partial responses are documented in the literature in approximately 10 to 23% of patients treated with imatinib. Our patient has been treated with imatinib for 24 months. The mean follow-up time in the literature varied from 12 to 19.7 months [7, 11]. However, not all clinical studies have been entirely positive regarding the use of imatinib. The French sarcoma group, in a SARC trial, demonstrated positive initial results of nonprogression rates at 3 and 6 months of 90% and 80%, respectively, but these decreased at 12 months to 67%. The median time to progression was 25 months in this study [6]. These results were confirmed in the SARC trial with initial progression-free survival of 94% and 88% at 1- and 2-months follow-up appointments but these decreased significantly to 66% at 1 year [12]. A 2012 review [13] reported the increasing interest in the potential role for tyrosine kinase inhibitors in the treatment of extra-abdominal desmoid tumors despite a limited role for imatinib alone but recommended it as part of therapeutic options. Sunitinib, another tyrosine kinase inhibitor, could be useful in some cases of AF, especially if there is resistance to imatinib [11]. Other systemic therapies, including nonsteroidal anti-inflammatory drugs such as indomethacin and sulindac, and tranilast, were used without randomized trials.

## Conclusions

The aggressiveness of desmoid tumors is related to their frequent recurrences despite surgical resection with clear margins. Surgery of tumors located in the subscapularis is almost R1 (resection is narrowed or involved microscopically) and recurrences or progression are evident. Systemic treatment with imatinib for unresectable or recurrent tumors with positive c-KIT could be the best therapeutic option. In our case report, the patient was stabilized with imatinib for 30 months and had a very good quality of life.

## Consent

Written informed consent was obtained from the patient for publication of this case report and accompanying images. A copy of the written consent is available for review by the Editor-in-Chief of this journal.

## Abbreviations

AF: Aggressive fibromatosis
*BCR-ABL*: Breakpoint cluster region-abelson gene
MRI: Magnetic resonance imaging
PDGFR: Platelet-derived growth factor receptor.
